# Prediction of silica nanoparticle biodistribution using a calibrated physiologically based model: Unbound fraction and elimination rate constants for the kidneys and phagocytosis identified as major determinants 

**DOI:** 10.5414/CP204837

**Published:** 2025-08-20

**Authors:** Madison Parrot, Joseph Cave, Maria Pelaez, Hamidreza Ghandehari, Prashant Dogra, Venkata Yellepeddi

**Affiliations:** 1 Division of Clinical Pharmacology, Department of Pediatrics, Spencer Fox Eccles School of Medicine,; 2 Department of Molecular Pharmaceutics, Utah Center for Nanomedicine, College of Pharmacy, University of Utah, Salt Lake City, UT,; 3 Mathematics in Medicine Program, Department of Medicine, Houston Methodist Research Institute, Houston, TX,; 4 Physiology, Biophysics, and Systems Biology Program, Graduate School of Medical Sciences, Weill Cornell Medicine, New York, NY,; 5 Applied Physics Graduate Program, Smalley-Curl Institute, Rice University, Houston, TX,; 6 Department of Biomedical Engineering, The John and Marcia Price College of Engineering, University of Utah, Salt Lake City, UT, and; 7 Department of Physiology and Biophysics, Weill Cornell Medical College, New York, NY, USA; *These authors contributed equally.

**Keywords:** silica nanoparticles, physiologically based pharmacokinetic modeling, pharmacokinetics, biodistribution, translation

## Abstract

Objectives: This study aimed to develop a minimal physiologically based pharmacokinetic (mPBPK) model to predict the biodistribution of silica nanoparticles (SiNPs) and evaluate how variations in surface charge, size, porosity, and geometry influence their systemic disposition. Materials and methods: The mPBPK model was calibrated using in vivo pharmacokinetic data from mice administered aminated, mesoporous, and rod-shaped SiNPs. Human data were collected from clinical trial data from Cornell dots. The mPBPK model incorporated physiological parameters and nanoparticle-specific characteristics to simulate SiNP biodistribution and was built in Matlab 2024a. Global sensitivity analysis identified influential parameters, including the unbound fraction and elimination rate constants for the kidneys and mononuclear phagocyte system (MPS). The model was extrapolated to predict human pharmacokinetics, with accuracy evaluated using Pearson correlation coefficients. Non-compartmental analysis (NCA) assessed organ-specific accumulation and biodistribution patterns. Results: Global sensitivity analysis revealed that the unbound fraction and elimination rate constants for the kidneys and MPS were major determinants of SiNP biodistribution. NCA indicated that aminated SiNPs initially accumulated in the liver, spleen, and kidneys but redistributed due to their high unbound fraction, while mesoporous SiNPs localized in the lungs. Rod-shaped SiNPs exhibited high lung exposure. The extrapolated model showed high predictive accuracy, with Pearson correlation coefficients of 0.98 for mice and 0.99 for humans. Conclusion: The mPBPK model effectively predicts the pharmacokinetics of diverse SiNPs, offering insights to optimize nanoparticle-based drug delivery systems and facilitating their translation from preclinical models to clinical applications.

**What is known about this subject**

- Silica nanoparticles are an important advancement in the field of drug delivery.
- The pharmacokinetics of silica nanoparticles have not been extensively explored.

**What this study adds**

- This physiologically based pharmacokinetic model describes the pharmacokinetics of silica nanoparticles in mice and humans.
- Physiologically based pharmacokinetic models assist in translation.

## Introduction 

In recent years, silica nanoparticles (SiNPs) have garnered notable attention due to their unique physical and chemical properties, making them valuable in drug delivery and imaging applications [1]. Their large surface area-to-volume ratio allows facile surface modification and conjugation, enhancing their utility in targeted drug delivery [2, 3]. For instance, functionalized SiNPs have demonstrated remarkable potential in imaging and targeted delivery to improve therapeutic efficacy and minimize side effects due to their tunable characteristics and biocompatibility [4, 5, 6, 7]. Despite their versatility, a consequential unmet need remains in translating these preclinical outcomes into clinical practice, as understanding their behavior in complex biological systems and addressing interspecies differences continue to pose challenges. 

SiNPs comprise of silicon dioxide (SiO_2_), a compound where silicon atoms are covalently bonded to oxygen atoms, forming a robust tetrahedral network and three-dimensional lattice structure [8]. This network of SiO_2_ can self-assemble into various nanostructures, including nanoparticles (NPs), with unique properties characterized by size, shape, porosity, and surface chemistry [8]. Among the various types of SiNPs, mesoporous SiNPs have potential as drug-delivery vehicles and diagnostics due to their adjustable pore size, tunable size, and geometry [9]. SiNPs can be included in gold or iron oxide composites for plasmonic and thermal ablation therapy [10, 11]. Biomedical applications of SiNPs have been extensively reviewed elsewhere [12, 13]. Common applications of SiNPs include drug delivery, diagnostics, and imaging. 

Pharmacokinetics (PK) studies how compounds are absorbed, distributed, metabolized, and excreted in biological systems. Knowledge of NP PK is essential for developing safe and effective NP-based therapies. Unlike traditional small molecules, NPs exhibit complex behaviors influenced by their size, geometry, surface properties, and unique interactions with biological components [14, 15, 16]. NPs are known to accumulate in the liver or spleen due to uptake by the resident macrophages in the sinusoidal blood vessels [17, 18]. Larger NPs, or those with a positive surface charge, show the highest uptake by the mononuclear phagocytic system (MPS) [19]. Kumar et al. [20] reviewed NP data from the literature to establish disposition trends and determined that SiNPs show higher lung, lower spleen, and lower heart concentrations than other NP types. 

The complexities of SiNPs necessitate advanced modeling approaches to predict NP PK accurately. PK modeling is a vital tool to understand NP behavior in vivo [21, 22]. The translation of NPs has only seen moderate success, highlighting the need for innovative methods in experimental design [23, 24, 25]. It has been established that NP characteristics do not consistently correlate with specific PK properties, particularly across NP types [26]. This necessitates using a PK modeling strategy for each NP type to establish the relationship between their physicochemical properties and PK parameters. 

Physiologically based pharmacokinetic (PBPK) models offer a sophisticated approach to understanding PK of therapeutics by incorporating detailed physiological and biochemical data [27, 28]. A whole-body PBPK model comprises several organ compartments where the compartments are connected via blood and lymph flow rates, with the transport phenomena mathematically characterized by a set of ordinary differential equations (ODEs). The ODEs contain known physiological parameters for the species of interest and unknown empirical parameters that are fitted using the observed data. These models simulate the whole-body behavior of therapeutics, providing insights into their PK profiles. PBPK models are widely utilized in drug development to bridge the gap between preclinical and clinical phases [29]. PBPK models find utility for optimizing the first-in-human dose to support the design of clinical trials [30]. 

PBPK models are particularly valuable for NPs, allowing researchers to account for their unique properties and biological interactions within the body. Despite the advancements in PBPK modeling, there remains a gap in the literature regarding models tailored explicitly for SiNPs. To our knowledge, no PBPK models of SiNPs in mice or humans are reported in the literature. We thus developed a PBPK model to evaluate SiNPs of different sizes, surface charges, and geometries for the first time. The primary objective of this study is to develop a minimal PBPK (mPBPK) model for six types of SiNPs in mice and extrapolate the animal model to humans to describe NP-specific characteristics that affect NP biodistribution. 

## Materials and methods 

### Preclinical and clinical NP PK data 

We obtained mice PK data for six silica NPs from a previously published study [31]. These NPs were either non-porous silica nanospheres (Stöber) or mesoporous SiO_2_. Specifically, they are mesoporous nanospheres (Meso), aminated mesoporous nanospheres (MA), Stöber nanospheres (Stöber), aminated Stöber nanospheres (SA), mesoporous nanorods with an aspect ratio of 8 (AR8), and aminated mesoporous nanorods with an aspect ratio of 8 (8A). The physicochemical characteristics of SiNPs investigated in this study are described in Table 1. The methods of NP synthesis are reported here [32]. All NPs were radiolabeled with ^125^I according to methods described previously [31]. The previous study also provides information on the characterization, surface modifications, and biodistribution data collection for the six SiNPs in mice [31]. Briefly, mice were injected with 20 mg/kg of NP suspension through the lateral tail vein. Blood and organs were harvested at 5 minutes, 30 minutes, 2 hours, 24 hours, and 72 hours. Radioactivity of the harvested organs was measured using a gamma counter and reported as the percent of injected dose per gram of tissue (%ID/g). Urine and feces were collected at 2, 24, 48, and 72 hours. Radioactivity from urine and feces was reported as the percentage of injected dose (%ID). The stability of the radioligand on SiNPs in mouse serum was confirmed for up to 72 hours using thin-layer chromatography (TLC). 

For clinical development of the model, we used human PK data for Cornell dots (^124^I-cRGDY–PEG–C dots), which are ultrasmall Stöber SiNPs modified with a peptide chain, polyethylene glycol (PEG), and ^124^I for positron emission tomography (PET) imaging. Methods of synthesis and characterization of Cornell dots (C dots) can be found in Phillips et al. [33]. Axial PET imaging for detection of the C dots was completed at 3, 24, and 72 hours after intravenous administration of 185 MBq (~ 3.4 to 6.7 nmol) for each human subject (n = 5). Radioactivity was reported as %ID/g. The size of the cRGDY–PEG–Cy5 coating was between 44 and 158 kDa [34]. The contribution to the overall weight of the radioactive tag to C dots was considered negligible. 

Plasma data for mice was digitized using PlotDigitizer 2.6.9 by Joseph A. Huwaldt (https://sourceforge.net/projects/plotdigitizer/files/plotdigitizer/). Organ data were obtained from the publication that reported the mice SiNP PK data [31]. Organ and plasma data for humans were obtained from the Cornell dot source paper [33]. 

### Model development 

**Figure 1.**
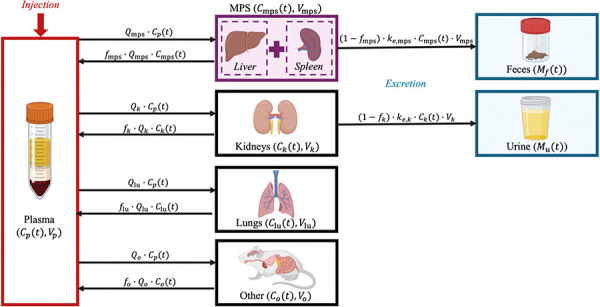
Illustration of the minimal physiologically based pharmacokinetic model to study the biodistribution and clearance of silica nanoparticles (SiNPs) across various compartments. This model quantifies SiNP concentrations in plasma (C_p_(t)), the mononuclear phagocyte system (MPS) (C_mps_(t)), lungs (C_lu_(t)), kidneys (C_k_(t)), and other tissues (C_0_(t)). Each compartment, indexed by i, is characterized by its blood flow rate (Q_i_), the fraction of unbound nanoparticles (f_i_), and volume (V_i_). The model also details nanoparticle excretion through feces (M_f_(t)) and urine (M_u_(t)), with clearance rates from the MPS (k_e,mps_) and kidneys (k_e,k_), respectively.

We developed a mPBPK model to simulate the whole-body biodistribution and clearance of SiNPs following intravenous injection. By emphasizing the biodistribution and clearance of SiNPs, which are directly influenced by their physicochemical properties, our semi-mechanistic model provides insights into how NP design affects their PK. The model includes seven key compartments: plasma, the MPS (comprising the liver and spleen), lungs, kidneys, urine, feces, and the rest of the body (“others”). A visual representation of the model is shown in Figure 1. 

A system of ODEs (Equations 1 – 7) was used to describe the concentration kinetics of SiNPs following intravenous administration. The model accounts for both perfusion-limited transport and first-order excretion processes. As shown in Figure 1, SiNPs are distributed to different organs based on their perfusion, which is determined by the blood flow rate Q_i_ for each organ i. The influx of SiNPs into each organ is proportional to the blood flow rate Q_i_ and the concentration of SiNPs in the plasma C_p_(t). The outflux is proportional to the concentration of freely circulating (i.e., unbound) SiNPs in the organ, represented by *f*_i_ × Ci(_t_), where C_i_(t) is the total concentration of SiNPs in organ i and *f*_i_ is the fraction of unbound SiNPs. This transport process is modeled as first-order, where both the influx and outflux are governed by perfusion-limited kinetics. 

The excretion of SiNPs occurs through the MPS and kidneys, both of which follow first-order excretion kinetics. The excretion term depends on the concentration of bound (i.e., non-circulating) SiNPs in each organ, modulated by an organ-specific excretion rate constant, k_e,i_. The fraction of bound SiNPs, 1−*f*_i_, is used to define the excretion rate as (1 – *f*_i_) × k_e,i_ × C_i_(t). In the MPS, SiNPs are excreted into feces, while in the kidneys, they are excreted into urine. 

The system of ODEs used to describe the concentration and mass kinetics of SiNPs in different compartments is as follows: 

### SiNP concentration kinetics in plasma 


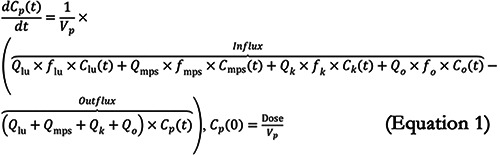


Where C_p_(t), C_lu_(t), C_mps_(t), C_k_(t), and C_o_(t) are the SiNP concentrations in plasma, lungs, MPS organs, kidneys, and other compartments, respectively; V_p_ is the volume of the plasma compartment; Q_lu_, Q_mps_, Q_k_, and Q_o_ represent the blood flow rates of lungs, MPS organs, kidneys, and other compartments, respectively; *f*_lu_, *f*_mps_, *f*_k_, and *f*_o_ are the fractions of freely circulating or unbound SiNPs in lungs, MPS organs, kidneys, and other compartments, respectively; is the injected dose of SiNPs. 

### SiNP concentration kinetics in MPS 


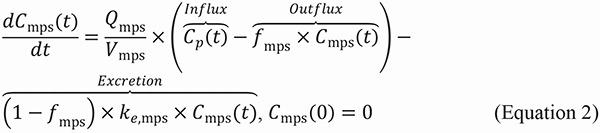


where V_mps_ is the volume of the MPS compartment, k_e,mps_ is the excretion rate constant from the MPS into the feces. 

### SiNP concentration kinetics in lungs 


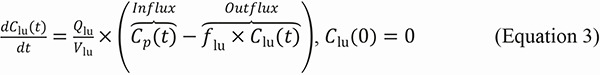


Where V_lu_ is the volume of the lungs compartment. 

### SiNP concentration kinetics in kidneys 


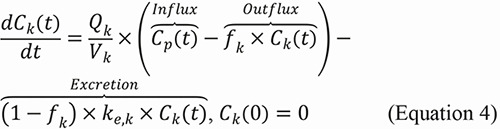


where V_k_ is the volume of the kidneys compartment; k_e,k_ is the excretion rate constant from the kidneys into the urine. 

### SiNP concentration kinetics in others 


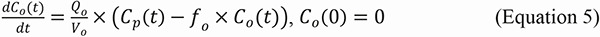


where V_o_ is the volume of the others compartment. 

### SiNP mass kinetics in urine 


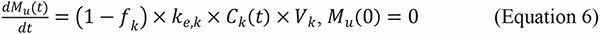


where M_u_(t) represents the mass of SiNPs excreted into the urine. 

### SiNP mass kinetics in feces 


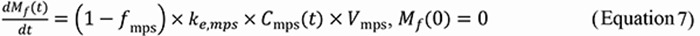


where M_f_(t) represents the mass of SiNPs excreted into the feces. 

### Numerical solution and parameter estimation 

The model was solved numerically as an initial value problem using MATLAB (version R2024a,MathWorks Inc., Natick, MA, USA). Initial conditions assume 100% of the injected dose in the plasma at time t = 0, with zero concentration in the remaining compartments. The system of ODEs was solved using *ode15s* solver, suitable for stiff systems. Eight key parameters were fitted based on observed experimental data: the fraction unbound in the lungs (*f*_lu_), kidneys (*f*_k_), MPS organs (*f*_mps_), and other compartments (*f*_0_), the excretion rate constants from the kidneys (k_e,k_) and MPS organs (k_e,mps_), and the blood flow rates for the MPS (Q_mps_) and other compartments (Q_0_). These parameters were fitted using non-linear least squares fitting via the *lsqcurvefit* function in MATLAB. The fitting process was constrained within physiological bounds, ensuring that parameters were non-negative, and the fraction unbound did not exceed 1. The remaining model parameters were obtained directly from literature (Table 2). 

### Interspecies scaling 

To assess the translational potential of our mPBPK model for predicting SiNP kinetics at the whole-body level, we extrapolated the mouse model to humans. Rate constants (k_e,k_ and k_e,mps_) were allometrically scaled based on established methods [35], using the following equation: 


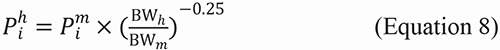


where P^h^_i_ and P^m^_i_ is the value of parameter for humans and mice, respectively; BW_h_ and BW_m_ is the body weight of humans (assumed to be 70 kg) and mice (assumed to be 0.02 kg), respectively. The standardized allometric scaling exponent for rate constants is –0.25 [36]. 

Note that the mouse physiological parameters for the model were obtained from the literature [37, 38, 39, 40, 41] and laboratory data (Table 2). Following allometric scaling, the human model parameters were recalibrated through least squares fitting using clinical data for C dots (Table 3) [31]. 

### Global sensitivity analysis 

Global sensitivity analysis (GSA) was performed for the mouse model to quantify the influence of the eight fitted model parameters on the model’s output, specifically the area under the curve (AUC) for each compartment (from 0 to 500 hours). GSA was conducted using Latin hypercube sampling (LHS), a statistical method that generates a sample of plausible parameter values from a multidimensional parameter distribution [42, 43]. In total, 1,000 parameter combinations were generated over 10 replicates using LHS, with parameters perturbed between ± 50% of their baseline values. Multivariate linear regression analysis (MLRA) was performed on each sample to generate sensitivity indices (SIs) for each model output, with the regression coefficients serving as the SIs. 

The parameters were then ranked based on the SIs using one-way ANOVA and Tukey’s test across ten replicates. The ranking identified the most influential parameters by examining the SI distribution for each parameter. A higher SI indicates a greater influence of the corresponding parameter on the model’s AUC outputs. This global approach was chosen over local sensitivity analysis, which only perturbs one parameter at a time, as it accounts for potential interactions and interdependencies between parameters. 

### Non-compartmental analysis 

Non-compartmental analysis (NCA) was performed using PKanalix version 2021R2 (Lixoft SAS, 2021, Antony, France, http://lixoft.com/products/PKanalix/) software. The AUC was calculated using the Linear Trapezoidal Linear method of PKanalix. The NCA was performed using plasma, MPS, lung, and kidney data. Reported data for mice and humans (in %ID/g units) was converted to concentration units for compatibility with PKanalix. The mean value for n = 5 mice is used for NCA due to the unavailability of individual mouse data; therefore, there is no error or standard deviation to report. 

## Results and discussion 

### Model development 

**Figure 2.**
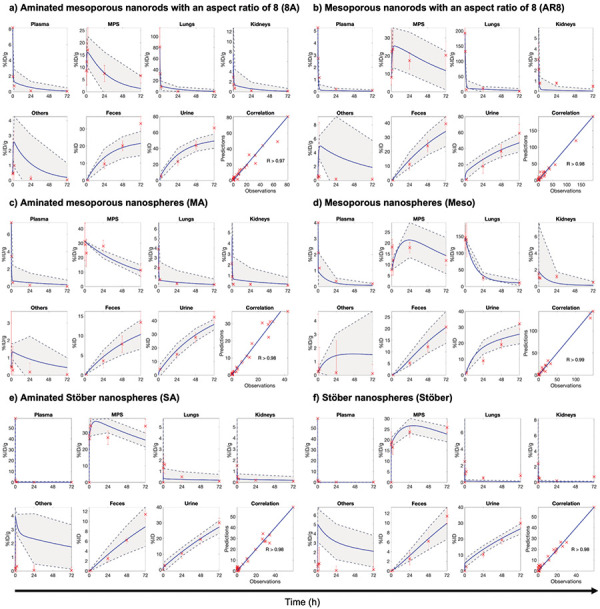
Minimal physiologically based pharmacokinetic model fits for various silica nanoparticles in mice. The temporal kinetics of nanoparticle (NP) concentration (%ID/g) in plasma, mononuclear phagocyte system (MPS), lungs, kidneys, and “others” compartment is shown for: a) mesoporous nanospheres (Meso), b) aminated mesoporous nanospheres (MA), c) Stöber nanospheres (Stöber), d) aminated Stöber nanospheres (SA), e) mesoporous nanorods with an aspect ratio of 8 (AR8), and f) aminated mesoporous nanorods with an aspect ratio of 8 (8A). Additionally, the cumulative mass kinetics (%ID) of NP excretion in urine and feces are shown for all NPs. Blue lines represent model fits, and red crosses with error bars represent the mean ± SD of in vivo data (n = 5 mice). The gray shaded areas correspond to the 95% confidence interval of the model fits. Goodness of fit is assessed by Pearson correlation coefficient (R), displayed for each fit. A common arrow marks the time axis across all plots but note that this time axis does not apply to the correlation plots. In vivo data was obtained from Yu et al. 2012 [31].

**Figure 3.**
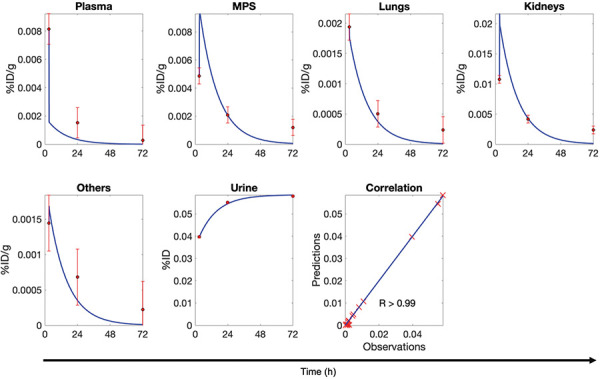
Minimal physiologically based pharmacokinetic model fits for Cornell dots (C dots) in humans. The temporal kinetics of nanoparticle (NP) concentration (%ID/g) in plasma, mononuclear phagocyte system (MPS), lungs, kidneys, and “others” compartment is shown for C dots. Additionally, the cumulative mass kinetics (%ID) of NP excretion in urine is shown. Blue lines represent model fits, and red circles with error bars represent the mean ± SD of clinical data (n = 5 subjects). Goodness of fit is assessed by Pearson correlation coefficient (R). A common arrow marks the time axis across all plots but note that this time axis does not apply to the correlation plots. Clinical data was obtained from Philips et al. 2014 [33].

We developed an mPBPK model to simulate the whole-body disposition kinetics of SiNPs with different physicochemical properties following intravenous administration in mice. The model was fitted to preclinical data for various SiNPs in mice [31] and clinical data for C dots in humans [33]. Allometric scaling was then used to extrapolate the mouse model to humans. As shown by the PK profiles of various SiNPs in mice (Figure 2) and C dots in humans (Figure 3), the model accurately fits the disposition of these NPs across seven compartments in mice and six compartments in humans. Model parameter estimates obtained through non-linear least squares fitting for both mice and humans are provided in Table 3. The Pearson correlation coefficients for the different types of mesoporous SiNPs – Meso, MA, Stöber, SA, AR8, and 8A – are 0.99, 0.98, 0.98, 0.98, 0.98, and 0.97, respectively. For the mean of the five human subjects, the Pearson correlation coefficient was 0.99, indicating the model’s strong agreement with the data. The experimental data consistently fell within the 95% confidence interval, underscoring the robustness and accuracy of the model in predicting NP biodistribution in essential organs for NPs with diverse physicochemical properties. While this model adopts a minimalistic approach, it successfully captures the essential dynamics, making it a valuable tool for preliminary assessments and guiding more detailed experimental designs. 

### Global sensitivity analysis 

**Figure 4.**
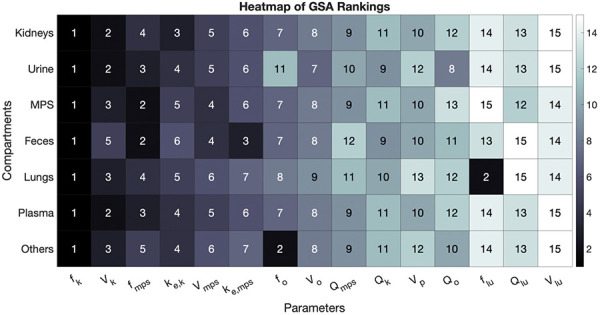
Global sensitivity analysis (GSA). Heat map of GSA-based rankings of various model parameters for their influence on the area under the curve (AUC) from 0 = 500 hours of nanoparticle (NP) concentration kinetics in the kidneys, plasma, lungs, mononuclear phagocyte system (MPS), and “others” compartments in mice. For the urine and feces compartments, the cumulative mass at the final time point was the model output of interest. Darker colors and lower numbers represent higher sensitivity indices, indicating greater parameter influence on the model output.

The GSA provided an in-depth examination of key model parameters influencing the biodistribution and retention of SiNPs across various compartments, including plasma, kidneys, MPS, lungs, and excretion pathways via urine and feces. Figure 4, a heatmap of GSA rankings, highlights the dominant role of certain parameters – particularly the unbound fraction in kidneys (*f*_k_) and MPS (*f*_mps_), as well as the elimination rate constants for kidneys (k_e,k_) and MPS (k_e,mps_) – in determining NP exposure within these compartments. The volume parameters (e.g., V_p_, V_mps_) were excluded from this analysis, as they are physiological constants and not influenced by NP physicochemical properties, making them less relevant to discussions on NP-specific biodistribution. 

The sensitivity indices for these parameters vary across compartments, as shown in Supplemental Figure S1, which presents violin plots for each compartment. Notably, the violin plots reveal the distribution of sensitivity indices, indicating that *f*_k_ consistently ranks as a top influencer in all compartments. Similarly, *f*_mps_ exerts a weighty effect across several compartments, reflecting the central role of MPS in mediating NP uptake and distribution, particularly for particles subject to phagocytosis. 

The model assumes that unbound fractions (*f*_k_, *f*_mps_) represent the proportion of NPs available for redistribution via blood flow, while the bound fractions (1−*f*_k_, 1−*f*_mps_) are subjected to elimination processes governed by the respective elimination rate constants (k_e,k_ and k_e,mps_). This assumption is corroborated by the GSA results, which show that higher unbound fractions (*f*) facilitate redistribution, thereby reducing compartment-specific retention. Conversely, lower unbound fractions lead to increased binding and retention within specific organs, as demonstrated by the high sensitivity of *f*_k_ and *f*_mps_ across relevant compartments. 

Additionally, the elimination rate constants k_e,k_ and k_e,mps_ exhibit marked sensitivity, especially in the kidneys and MPS, indicating that these rates substantially affect the clearance of bound NPs from these compartments. This relationship is further illustrated in Figure 4, where the kidneys and MPS show strong dependencies on their respective elimination rate constants. The violin plots in Supplemental Figure S1 support this finding, with distinct distributions of sensitivity indices for k_e,k_ and k_e,mps _across compartments, highlighting how variations in elimination dynamics contribute to overall SiNP biodistribution. 

Taken together, these GSA findings underscore the pivotal role of the fraction unbound (*f*) and elimination rate constants (k) in modulating NP exposure, distribution, and clearance across compartments. These parameters, as influenced by NP physicochemical properties such as shape, surface charge, and porosity, determine how NPs behave systemically and in specific organs. This foundational understanding facilitates the interpretation of AUC trends across compartments based on the interplay of NP properties with *f* and k parameters. These insights are instrumental in guiding NP design for optimized biodistribution and therapeutic efficacy, as explored in the subsequent sections. 

### In*f*luence o*f* NP physicochemical properties on biodistribution and exposure across compartments 

Analyzing exposure patterns across plasma, lungs, MPS, and kidneys reveals how SiNP physicochemical properties – such as shape, porosity, and surface charge – affect biodistribution. The general trend observed is a coupling effect between plasma and lung exposure, indicating that extended circulation in the plasma enhances lung accumulation across most NPs. Additionally, mass balance trends highlight compensatory distribution among compartments, where considerable accumulation in one compartment is countered by lower exposure in others, reflecting conservation of the SiNP mass within the system. 

**Plasma exposure**

**Figure 5.**
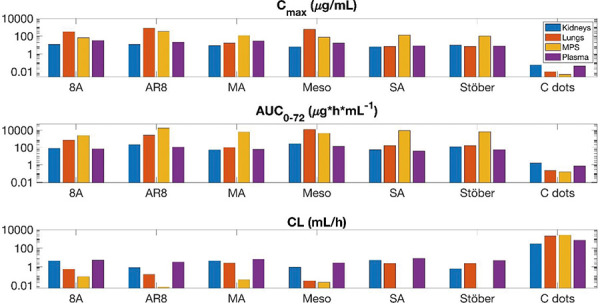
Non-compartmental analyses (NCA). Bar plot displaying results of NCA for each silica nanoparticle (SiNP), including the following pharmacokinetic (PK) parameters: maximum concentration (C_max_), area under the concentration curve from time 0 to the final recorded timepoint (AUC_0–72_), and clearance (CL) in the plasma, lungs, mononuclear phagocyte system (MPS) organs, and kidney compartments.

Among the six SiNPs, Meso exhibits the highest plasma AUC (130 µg×h/mL) (Figure 5), attributable to its mesoporous structure, moderate unbound fraction (*f*_k_ = 0.513) (Table 3), and lower elimination rate (k_e,k_ = 3.357 h^−1^). This extended plasma exposure indicates that Meso is well-retained in circulation, enabling prolonged systemic availability. MA and SA have notably lower plasma AUCs (60 and 38 µg×h/mL, respectively), reflecting their higher unbound fractions (*f*_k_ = 0.926 and *f*_k_ = 0.747) and rapid redistribution, which reduces overall retention. Non-porous Stöber also shows limited plasma exposure (58 µg×h/mL), which could stem from its structural rigidity and moderate unbound fraction (*f*_k_ = 0.541). In comparison, AR8 demonstrates a relatively higher plasma AUC (110 µg×h/mL) due to its rod shape and mesoporous structure, balancing systemic circulation and selective organ retention. 

**Lungs exposure**

A coupling effect between plasma and lung exposure is evident across most SiNPs, as observed with Meso and AR8. Meso has the highest lung AUC (11,790 µg×h/mL) (Figure 5), facilitated by its low unbound fraction (*f*_mps_ = 0.005) (Table 3) and moderate elimination rate (k_e,mps_ = 0.010 h^−1^), which supports prolonged retention. AR8 also displays substantial lung exposure (2,590 µg×h/mL), likely due to its rod shape and mesoporous structure, enabling enhanced interaction with the alveolar surface. In contrast, non-porous NPs Stöber and SA have lower lung AUCs (160 µg×h/mL each), suggesting that their structure limits lung retention despite low unbound fractions. MA, with the highest unbound fraction (*f*_mps_ = 0.497), has the lowest lung AUC (100 µg×h/mL), indicating that increased unbound fractions enhance systemic redistribution, reducing localized retention in the lungs. 

**MPS exposure**

AR8 has the highest MPS exposure (17,200 µg×h/mL) (Figure 5), reflecting a strong affinity for macrophage-rich organs such as the liver and spleen. This high retention is supported by its rod shape and mesoporous structure, which likely enhance interactions with macrophages, coupled with a low unbound fraction (*f*_mps_ = 0.003) and moderate elimination rate (k_e,mps_ = 0.016 h^−1^). These features enable AR8 to remain bound and accumulate within the MPS, effectively aligning its structural and pharmacokinetic characteristics with enhanced macrophage uptake. SA also shows high MPS exposure (8,520 µg×h/mL) due to a similarly low elimination rate (k_e,mps_ = 0.0025 h^−1^) and non-porous structure, which likely enhances retention within the liver and spleen. Stöber and MA exhibit moderate MPS exposure (6,450 and 6,030 µg×h/mL, respectively) (Figure 5). The non-porous structure and distinct surface charges of these particles (negative for Stöber and positive for MA) influence their interactions within the MPS. Stöber has a relatively low unbound fraction (*f*_mps_ = 0.002) and low elimination rate constant (k_e,mps_ = 0.003 h^−1^), promoting some retention within the MPS but to a lesser extent than highly retained particles. In contrast, MA, with a considerably higher unbound fraction (*f*_mps_ = 0.497) and similar elimination rate (k_e,mps_ = 0.009 h^−1^), redistributes more readily, resulting in moderate MPS accumulation. The high MPS retention of these SiNPs, contrasted with their moderate kidney and lung exposure, suggests a compensatory mass balance effect, where predominant uptake in the MPS is coupled with lower distribution in other compartments. 

**Kidneys exposure**

In the kidneys, Meso displays moderate exposure (AUC = 270 µg×h/mL) (Figure 5), facilitated by a balanced unbound fraction (*f*_k_ = 0.513) and low elimination rate, allowing retention without rapid clearance. AR8 shows slightly lower kidney exposure (AUC = 210 µg×h/mL), with its rod shape and mesoporous structure promoting selective retention in MPS and lungs rather than renal accumulation. Stöber exhibits reduced kidney retention (AUC = 120 µg×h/mL), with a moderate unbound fraction and higher elimination rate favoring redistribution. In contrast, amine-coated MA and SA exhibit even lower kidney exposures (52 and 55 µg×h/mL, respectively), driven by their high unbound fractions, which enhance circulation and systemic redistribution rather than local retention in the kidneys. 

The analysis across compartments reveals a general coupling effect between plasma and lung exposure, with mesoporous NPs such as Meso and AR8 achieving substantial retention in both compartments. This coupling is likely a function of their structural properties, which enhance circulation and lung accumulation simultaneously. In contrast, non-porous NPs (Stöber and SA) and amine-coated NPs (MA and 8A) display lower lung exposure, attributed to their higher unbound fractions that promote rapid redistribution rather than compartmental retention. 

The mass balance pattern seen across compartments suggests a conservation of the SiNP mass within the system, where high MPS exposure in particles like AR8 and SA coincides with reduced exposure in plasma and kidneys, indicating a preferential uptake by macrophage-rich organs. This mass balance insight emphasizes that each NP’s physicochemical profile influences its distribution, where mesoporous and rod-shaped NPs are more prone to lung and MPS retention, while non-porous and highly charged NPs favor circulation with minimal organ-specific retention. 

Overall, these findings underscore the importance of optimizing NP characteristics – such as porosity, shape, and surface charge – to achieve targeted exposure profiles. The coupling effect and mass balance patterns across plasma, lungs, MPS, and kidneys provide a basis for designing NPs tailored to desired biodistribution and retention profiles, advancing therapeutic efficacy and minimizing off-target effects. 

### Non-compartmental analysis 

The results of the NCA for mice are shown in Table 4 and for humans in Table 5. It should be noted that clearance-related results of the NCA for the MPS compartment with Stöber NPs are unavailable due to an observed accumulation trend in MPS organs. As a result of the NCA, patterns in the PK of SiNPs were established based on the physical characteristics of SiNPs, including surface charge, size, and geometry. A visual representation in the form of a heat map of the SiNPs and the respective PK trend is shown in Figure 5. 

The aminated SiNPs (8A, SA, and MA) tend to have the highest maximum concentration (C_max_), area under the concentration curve from zero to infinity (AUC_0–∞_), and lowest clearance (CL) in the MPS and kidneys. Rod-shaped NPs tend to have a higher C_max_ in the lungs and plasma. MA and 8A SiNPs with the same highly positive zeta potential (20 – 40 mV), show the same magnitude C_max_, AUC_0–∞_, and CL in the plasma. 

Since the process of extravasation involves the SiNPs moving from the bloodstream into the tissue, highly protein-bound SiNPs will have limited extravasation. Similarly, only unbound SiNPs can be recognized and internalized by macrophages. This will affect the clearance of the SiNPs as those with a higher fraction unbound will have higher uptake by the liver and spleen. For example, larger SiNPs have been established to reduce alveolar macrophage uptake [44]. Overall, capturing the unbound fraction via in vitro experiments would be necessary for a more meticulous model of NPs, including extravasation- and phagocytosis-dependent biodistribution and clearance. 

The size and surface modification of SiNPs have been established to affect immune response [45, 46]. Small NPs are more likely to undergo phagocytosis and be cleared by the reticuloendothelial system (RES) [47]. Smaller NPs have a higher surface area-to-volume ratio than larger particles. This increased surface area allows for more interactions with biological fluids, including plasma proteins and components of the immune system. Small NPs may pass through renal corpuscle cut-off (10 nm) [48], leading to increased, even rapid, clearance through the urine [49]. NPs with diameters exceeding this threshold are retained longer in the plasma due to decreased renal filtration. Interaction with renal tubules, which reabsorb water and nutrients, affects the excretion rates of NPs and their potential accumulation in the kidneys. Evidently, rapid clearance leads to reduced accumulation in organ tissues. This phenomenon is evidenced by the lowest AUC_0–∞_ and C_max_ and the quickest clearance of the C dots in all organs explored (plasma, lungs, MPS, kidney). This is supported by other NP studies that observed that other NPs have higher cellular uptake than smaller ones [50, 51]. The surface charge of the C dots administered in the source literature for this model was not reported. However, the surface conjugation of Cy5 dye contains negative sulfonate groups that will impart a negative charge. 

Lung accumulation is observed with Meso and AR8 particles, two mesoporous SiNPs that are not amine-modified. AR8 and Meso have the highest AUC_0–∞_ and C_max_ and lowest CL in the lungs, likely due to their negative zeta potential (–40 to –30 mV). Amine modification reduces macrophage uptake in the lung. It reduces the risk of lung injury due to reduced induction of endosomal reactive oxygen species (ROS), leading to fewer ROS-dependent pathways for proinflammatory species induction [44]. It has been well documented that SiNPs of mesoporous nature preferentially accumulate in the lungs due to the lungs’ vascular nature, permeability, and capacity to retain substances and has led to an investigation of their use as drug delivery and imaging agents for lung-related ailments [52, 53, 54, 55]. 

Despite Stöber having a lower zeta potential than Meso and AR8 (–60 to –40 mV), lung accumulation is not observed. Notably, the non-porous version of Meso, Stöber, and Stöber’s aminated counterpart, SA, does not exhibit lung accumulation but instead accumulates in the MPS organs. The PK patterns of Stöber illustrate the complexity of PK when multiple physical properties are involved. 

The MPS plays a critical role in the plasma dynamics of NPs. Parameters such as k_e,mps_ and *f*_mps_ directly influence NP levels through mechanisms of binding, uptake, and excretion. Upon entry into the bloodstream, NPs quickly bind to plasma proteins, forming a dynamic protein corona that modifies their biological identity and influences biodistribution and clearance. Coronas enriched with opsonins enhance phagocytic clearance by MPS cells. In organs like the liver and spleen, specialized endothelial structures and macrophages facilitate the capture and processing of NPs, leading to their excretion or degradation. A high k_e,mps_ accelerates NP clearance from plasma, whereas a low k_e,mps_ permits longer systemic circulation. The blood flow to MPS organs, while substantial, is typically not a limiting factor in NP clearance, assuming that perfusion is sufficient for effective NP delivery. 

The mPBPK model was successfully created and verified with in vivo mouse and human biodistribution data. The Pearson correlation coefficient attests to a high level of agreement between the model predictions and observations. To our knowledge, only one other SiNP PBPK model has been created that was specific to rats [43]. Other PBPK models for NPs have been created in MATLAB [56, 57, 58, 59]. The current model does not include NP-specific parameters such as size, zeta potential, or geometry. We can establish patterns in biodistribution based on these parameters post-modeling. This study quantified the SiNPs in plasma and tissues using an indirect radioactive label-based method. However, in our future studies, we would like to evaluate the PK of SiNPs and develop the PBPK model using PK data from a direct method of analysis using inductively coupled plasma mass spectrometry (ICP-MS). 

## Conclusion 

This work successfully developed a minimal PBPK model for SiNPs, integrating biodistribution data from both human and mouse subjects, with robust validation demonstrated through high Pearson correlation coefficients. The model highlighted critical parameters – such as unbound fraction in kidneys (*f*_k_) and MPS (*f*_mps_), alongside kidney and MPS elimination rate constants (k_e,k_ and k_e,mps_) – that highly influence NP exposure across compartments, underscoring the roles of protein binding, extravasation, and macrophage uptake in biodistribution. Additionally, the effects of NP physicochemical characteristics, including shape, porosity, and surface charge, were evident in exposure patterns, such as the plasma-lung coupling seen with certain mesoporous NPs and preferential MPS retention in macrophage-rich organs. 

Future work will refine this model by incorporating direct ICP-MS analysis for precise PK data and expanding the model to account for drug delivery applications via SiNPs. This approach will enhance the model’s applicability and predictive accuracy for NP behavior in various biological systems, supporting its potential utility in dosing optimization, targeted organ delivery, and regulatory safety evaluations. Ultimately, this reduced model serves as a powerful tool to bridge preclinical and clinical findings, driving forward the safe and effective use of SiNP-based therapeutics in translational medicine. A robust PBPK model for SiNPs will significantly advance the field of nanomedicine by providing valuable insights into their PK in humans. In conclusion, this research successfully developed a PBPK model for SiNPs by integrating biodistribution data from both human and mouse subjects. The model highlighted key factors influencing NP behavior, including the role of protein binding in extravasation and phagocytosis and the impact of NP size and surface modifications on biodistribution and clearance. Future work will refine this model by incorporating direct ICP-MS analysis and exploring the delivery of drugs via SiNPs, enhancing its applicability and accuracy for predicting NP behavior in various biological systems. This research has the potential to impact the translational success of drug development, safety assessments, and regulatory evaluations of NP-based therapeutics, ultimately contributing to the safe and effective use of these innovative therapies. 

## Acknowledgment 

PD and JC acknowledge Carmine Schiavone for helpful scientific discussions. 

## Data availability 

The datasets are available from the corresponding author on reasonable request. 

## Authors’ contributions 

VY and PD conceived the study. PD developed the model and designed the analysis. MP, JC, MJP, PD, and VY performed the analysis. PD, HG, and VY interpreted the results. MP, JC, VY, and PD wrote the manuscript. MJP, and HG edited the manuscript.

## Funding 

This research was supported by NIH grant R03EB033576 (PI – VY, CO-Is – PD and HG). The funders had no role in study design, data collection and analysis, publication decision, or manuscript preparation. 

## Conflict of interest 

The authors declare no conflict of interest. 

**Table 1.** Nanoparticle (NP) characterization. The NPs evaluated are mesoporous nanospheres (Meso), aminated mesoporous nanospheres (MA), Stöber nanospheres (Stöber), aminated Stöber nanospheres (SA), mesoporous nanorods with an aspect ratio of 8 (AR8), aminated mesoporous nanorods with an aspect ratio of 8 (8A), and Cornell dots (C dots). Surface charge is ranked as highly negative (−60 to −40 mV; symbol ---), moderately negative (−40 to −30 mV; symbol --), moderately positive (+10 to +20 mV; symbol ++), highly positive (+20 to +40 mV; symbol +++).

NP type	NP size by TEM (nm)	Surface charge	Surface modification	Porosity	Shape
Meso	120	--	None	Mesoporous	Sphere
MA	120	+++	Amine	Mesoporous	Sphere
Stöber	115	---	None	Non-porous	Sphere
SA	115	++	Amine	Non-porous	Sphere
AR8	136 x 1,028	--	None	Mesoporous	Rod
8A	136 x 1,028	+++	Amine	Mesoporous	Rod
C dots	6	N/A	cRGDY peptides, PEG, Cy5	Non-porous	Sphere

TEM = transmission electron microscopy; N/A = not available.

**Table 2.** Physiological parameter values used in the physiologically based pharmacokinetic model.

Parameter (units)	Mouse	Human
Heart weight (g)	0.162^b^	310^a^
Liver weight (g)	1.45^b^	1690^a^
Spleen weight (g)	0.1166^b^	192^a^
Lungs weight (g)	0.515^b^	1170^a^
Kidneys weight (g)	0.496^b^	280^a^
Brain weight (g)	0.474^b^	1450^a^
Stomach weight (g)	0.587^b^	N/A
Small intestine weight (g)	1.28^b^	N/A
Large intestine weight (g)	0.55^b^	N/A
Tail weight (g)	0.018^b^	N/A
Carcass weight (g)	10.2^a^	N/A
GI tract weight (g)	N/A	1,650^a^
Muscle weight (g)	N/A	35,000^a^
Parotid weight (g)	N/A	27.5^d^
Thyroid weight (g)	N/A	16.4^e^
Volume of plasma (mL)	1.7^a^	3,000^a^
V_mps_; volume of MPS (mL)	1.56^f^	1,882^f^
V_lu_; volume of lungs (mL)	0.514^f^	1,170^f^
V_k_; volume of kidneys (mL)	0.496^f^	280^f^
V_0_; volume of others (mL)	13.2^f^	38,453.9^f^
Blood flow through lungs (mL/h)	667^b^	330,000^c^
Blood flow through MPS (mL/h)	113.4^a^	87,000^a^
Blood flow through kidneys (mL/h)	78^a^	74,400^a^
Blood flow through others (mL/h)	60^b^	167,400^b^

^a^Davies and Morris et al., Pharm Res. (1993); ^b^Tian et al. 2012; ^c^Ivanov et al., Bull Exp Biol Med. (2013); ^d^De Ferraris et al., Médica panamericana. (1999); ^e^Pankow et al., Health Phys. (1985). ^f^Organ volumes were equal to their corresponding weights based on the assumption of organ density to be 1 g/mL. N/A = parameter is not applicable to the respective model.

**Table 3.** Minimal physiologically based pharmacokinetic model parameter estimates from least squares regression.

Parameter	Definition (units)	Mice						Humans
		8A	AR8	MA	Meso	SA	Stöber	C dots
*f*_lu_	Unbound fraction of NPs in lungs (n/a)	0.121	0.011	0.999	0.006	0.718	0.478	0.889
*f*_mps_	Unbound fraction of NPs in MPS (n/a)	0.073	0.003	0.497	0.005	0.0057	0.002	0.155
*f*_k_	Unbound fraction of NPs in kidneys (n/a)	0.907	0.596	0.926	0.513	0.747	0.541	0.078
*f*_0_	Unbound fraction of NPs in others (n/a)	0.541	0.029	0.508	0.065	0.083	0.019	0.964
*Q*_mps_	Blood flow rate in MPS (mL/h)	4.17	19.0	0.032	3.08	60.9	35.6	80,854
*Q*_0_	Blood flow rate in others (mL/h)	9.56	43.492	6.962	1.649	76.07	84.858	165,380
*k*_e,mps_	Excretion rate constant in the MPS (h^–1^)	0.032	0.016	0.009	0.01	0.0025	0.003	0.452
*k*_e,k_	Excretion rate constant in the kidneys (h^–1^)	27.3	21.7	32.6	3.4	12.2	15.4	2.67e-4

MPS = mononuclear phagocyte system.

**Table 4.** Non-compartmental analysis (NCA) of the disposition kinetics data for various silica nanoparticles (SiNPs) in mice. The maximum concentration (C_max_), time to maximum concentration (t_max_), area under the concentration curve from zero to infinity (AUC_0–∞_), area under the concentration curve from zero to seventy-two hours (AUC_0–72_), clearance (CL), volume of distribution at steady state (V_d,ss_), and half-life (T_1/2_) were calculated.

NP type	PK parameters	Plasma	Lungs	MPS	Kidneys
8A	C_max_ (µg/mL)	33	320	66	12
	t_max_ (h)	0	0	1.92	0
	AUC_0–∞_ (h×µg×mL^–1^)	76	720	4540	91
	AUC_0–72_ (h×µg×mL^–1^)	71	710	2430	78
	CL (mL/h)	5.24	0.56	0.088	4.41
	V_d,ss_ (mL)	123.42	7.63	7.38	136.27
	T_1/2_ (h)	16.5	9.49	57.75	21.66
AR8	C_max_ (µg/mL)	21	770	330	12
	t_max_ (h)	0	0	0.42	0
	AUC_0–∞_ (h×µg×mL^–1^)	120	2,680	60,050	490
	AUC_0–72_ (h×µg×mL^–1^)	110	2,590	17,200	210
	CL (mL/h)	3.3	0.15	0.0067	0.82
	V_d,ss_ (mL)	84.43	2.72	1.31	114.23
	T_1/2_ (h)	17.76	43.31	135.88	96.25
MA	C_max_ (µg/mL)	29	16	120	8.4
	t_max_ (h)	0	0	0	0
	AUC_0–∞_ (h×µg×mL^–1^)	61	150	9,600	90
	AUC_0–72_ (h×µg×mL^–1^)	60	100	6,030	52
	CL (mL/h)	6.59	2.61	0.042	4.45
	V_d,ss_ (mL)	104.88	159.23	3.4	418.7
	T_1/2_ (h)	11	43.3	216.5	63
Meso	C_max_ (µg/mL)	16	590	73	6
	t_max_ (h)	0	0.42	0.42	0
	AUC_0–∞_ (h×µg×mL^–1^)	150	12,950	19,040	440
	AUC_0–72_ (h×µg×mL^–1^)	130	11,790	4,290	270
	CL (mL/h)	2.66	0.031	0.021	0.91
	V_d,ss_ (mL)	85.57	0.84	6.49	77.22
	T_1/2_ (h)	22.35	18.72	216.56	57.75
SA	C_max_ (µg/mL)	8.2	6.8	130	6
	t_max_ (h)	0	0.42	1.92	0
	AUC_0–∞_ (h×µg×mL^–1^)	49	170	N/A	81
	AUC_0–72_ (h×µg×mL^–1^)	38	160	8520	55
	CL (mL/h)	8.08	2.32	N/A	4.96
	V_d,ss_ (mL)	344.76	58.61	N/A	320.02
	T_1/2_ (h)	30.13	17.32	N/A	46.2
Stöber	C_max_ (µg/mL)	7.5	6.8	100	9.6
	t_max_ (h)	0	0.42	71.92	0
	AUC_0–∞_ (h×µg×mL^–1^)	83	170	N/A	640
	AUC_0–72_ (h×µg×mL^–1^)	58	160	6,450	120
	CL (mL/h)	4.84	2.32	N/A	0.62
	V_d,ss_ (mL)	186.58	58.61	N/A	115.46
	T_1/2_ (h)	26.65	17.32	N/A	128.33

**Table 5.** Non-compartmental analysis (NCA) of the disposition kinetics data for Cornell dots in humans. The maximum concentration (C_max_), time to maximum concentration (t_max_), area under the concentration curve from zero to infinity (AUC_0–∞_), area under the concentration curve from zero to 72 hours (AUC_0–72_), clearance (CL), volume of distribution at steady state (V_d,ss_), and half-life (T_1/2_) were evaluated.

PK parameters	Plasma	Lungs	MPS	Kidneys
C_max_ (µg/mL)	0.042 ± 0.0061	0.0099 ± 0.0021	0.0049 ± 0.0014	0.055 ± 0.066
t_max_ (h)	0	0	0	0
AUC_0–∞_ (h×µg×mL^–1^)	0.77 ± 0.22	0.31 ± 0.19	0.22 ± 0.08	2.67 ± 1.56
AUC_0–72_ (h×µg×mL^–1^)	0.74 ± 0.19	0.22 ± 0.038	0.15 ± 0.047	1.6 ± 0.96
CL (mL/h)	709.97 ± 222.66	2 013.41 ± 778.9	2,617.02 ± 972.06	296.3 ± 252.57
V_d,ss_ (mL)	13,981.34 ± 2,716.1	62,583.72 ± 13,919.87	134,348 ± 42,236.39	16,796.2 ± 7,882.2
T_1/2_ (h)	14.31 ± 3.54	28.42 ± 24.14	31.71 ± 6.09	51.81 ± 33.67

MPS = mononuclear phagocyte system.

## Supplemental material

Supplemental material Supplemental Figure S1
