# Supplementary material for: Prediction of silica nanoparticle biodistribution using a calibrated physiologically based model: Unbound fraction and elimination rate constants for the kidneys and phagocytosis identified as major determinants
Source: Int J Clin Pharmacol Ther. 2025 Aug 20;63(12):584–600. doi: 10.5414/CP204837 (PMC12825016; doi:10.5414/CP204837)

Figure S1. Violin plot of GSA-based rankings for the various model parameters in different model compartments of mice.

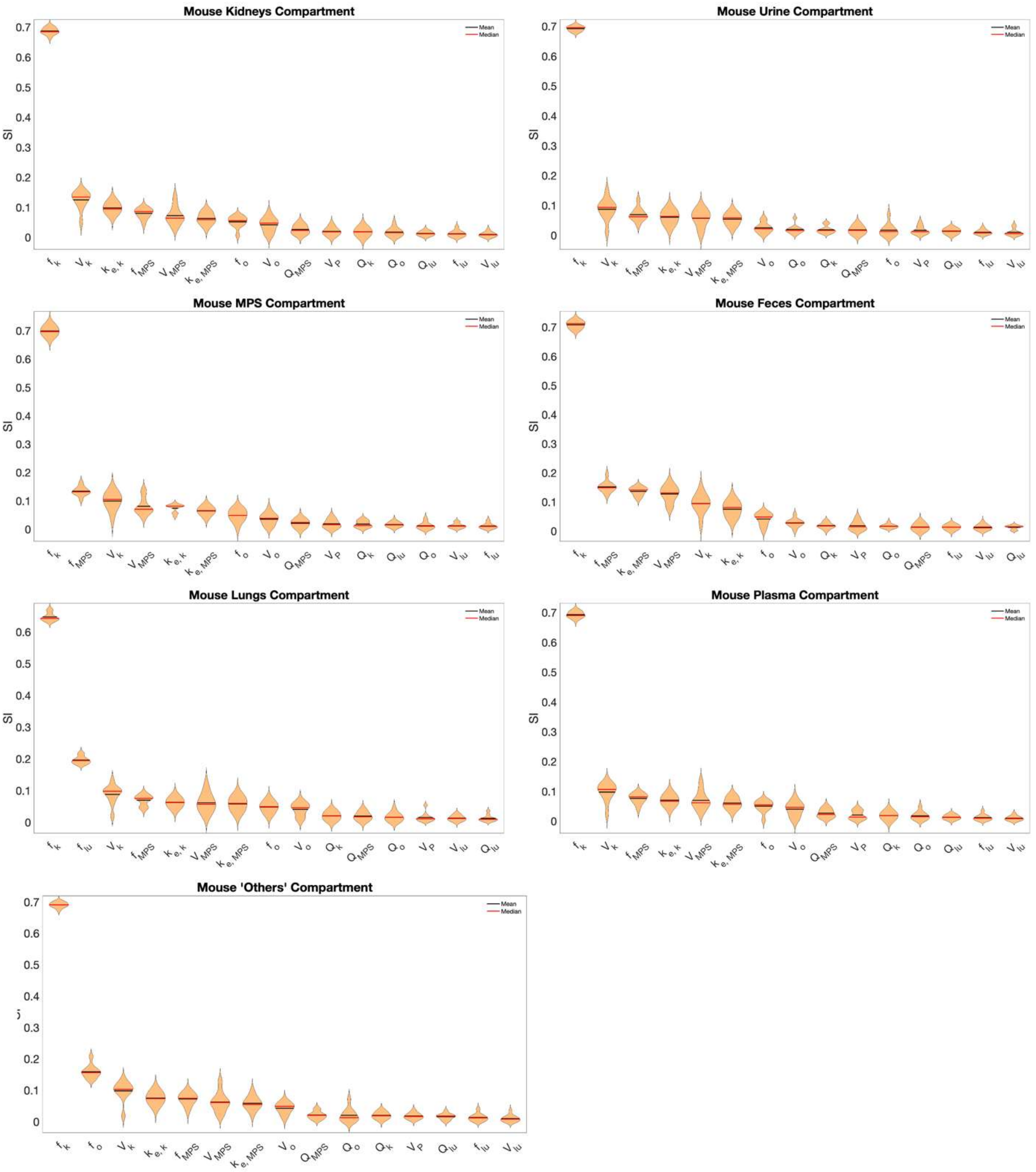

Supplement: Supplemental material [file intjclinpharmacol-63-584-S01.pdf]
